# DISC1-dependent Regulation of Mitochondrial Dynamics Controls the Morphogenesis of Complex Neuronal Dendrites*

**DOI:** 10.1074/jbc.M115.699447

**Published:** 2015-11-09

**Authors:** Rosalind Norkett, Souvik Modi, Nicol Birsa, Talia A. Atkin, Davor Ivankovic, Manav Pathania, Svenja V. Trossbach, Carsten Korth, Warren D. Hirst, Josef T. Kittler

**Affiliations:** From the ‡ Department of Neuroscience, Physiology, and Pharmacology, University College London, Gower Street, London WC1E 6BT, United Kingdom,; the ¶ Neuroscience Research Unit, Pfizer, Cambridge, Massachusetts 02139, and; the § Department of Neuropathology, Heinrich Heine University, Moorenstrasse 5, 40225 Dusseldorf, Germany

**Keywords:** dendrite, endoplasmic reticulum (ER), mitochondria, schizophrenia, trafficking, DISC1, DISC1-Boymaw fusion protein, Miro, mitofusin

## Abstract

The DISC1 protein is implicated in major mental illnesses including schizophrenia, depression, bipolar disorder, and autism. Aberrant mitochondrial dynamics are also associated with major mental illness. DISC1 plays a role in mitochondrial transport in neuronal axons, but its effects in dendrites have yet to be studied. Further, the mechanisms of this regulation and its role in neuronal development and brain function are poorly understood. Here we have demonstrated that DISC1 couples to the mitochondrial transport and fusion machinery via interaction with the outer mitochondrial membrane GTPase proteins Miro1 and Miro2, the TRAK1 and TRAK2 mitochondrial trafficking adaptors, and the mitochondrial fusion proteins (mitofusins). Using live cell imaging, we show that disruption of the DISC1-Miro-TRAK complex inhibits mitochondrial transport in neurons. We also show that the fusion protein generated from the originally described DISC1 translocation (DISC1-Boymaw) localizes to the mitochondria, where it similarly disrupts mitochondrial dynamics. We also show by super resolution microscopy that DISC1 is localized to endoplasmic reticulum contact sites and that the DISC1-Boymaw fusion protein decreases the endoplasmic reticulum-mitochondria contact area. Moreover, disruption of mitochondrial dynamics by targeting the DISC1-Miro-TRAK complex or upon expression of the DISC1-Boymaw fusion protein impairs the correct development of neuronal dendrites. Thus, DISC1 acts as an important regulator of mitochondrial dynamics in both axons and dendrites to mediate the transport, fusion, and cross-talk of these organelles, and pathological DISC1 isoforms disrupt this critical function leading to abnormal neuronal development.

## Introduction

The disrupted in schizophrenia 1 (DISC1) protein is a promising candidate susceptibility factor for major mental illness (1). Multiple genetic studies have shown an association between DISC1 and schizophrenia, bipolar disorder, major depression, and autism (2, 3). DISC1 was first discovered due to a balanced chromosomal translocation in a family with a high incidence of schizophrenia and other major mental illness (4). This translocation results in the truncation of *DISC1* after exon 8 and the fusion to another gene, *Boymaw* (also known as *DISC1FP1* for DISC1 fusion partner 1), leading to the expression of a DISC1-Boymaw fusion protein (5, 6). DISC1 affects multiple cellular functions including neuronal proliferation, migration, and integration via its roles at the centrosome in the anchoring of key proteins such as Bardet-Biedl syndrome (BBS) proteins BBS1 and BBS4 (7). DISC1 also regulates intracellular signaling pathways such as the Wnt/β-catenin and PDE4 signaling pathways (8, 9) and regulates neurite outgrowth. Point mutations or truncation of DISC1 leads to decreased dendritic complexity, both *in vivo* and in dissociated culture (10–12), highlighting the necessity for normal DISC1 function in neuronal development. However, the mechanisms by which DISC1 contributes to altered neuronal development, function, and pathology remain poorly understood. Moreover, the cellular impact of expression of the Boymaw fusion protein also remains unclear.

Mitochondria are highly dynamic organelles that undergo constant trafficking, fission, fusion, and turnover. In neurons, the tight regulation of mitochondrial transport is critical to allow controlled delivery of these organelles to sites where they are required for energy provision and calcium buffering (13). Disruption of mitochondrial localization can lead to defects in synaptic function and plasticity in addition to affecting neuronal morphology (13, 14). Detailed studies have revealed mitochondrial distribution and bidirectional trafficking to be regulated in a calcium-dependent manner via the mitochondrial Rho GTPases Miro1 and Miro2 (15–19, 78). These outer mitochondrial membrane proteins possess two calcium-sensing EF-hand domains flanked by two GTPase domains on their cytoplasmic face (20, 21). Miro1 interacts with kinesin and dynein motors and their TRAK adaptor proteins (22–24). TRAK1 has been recently demonstrated to be axonally targeted, whereas TRAK2 favors a dendritic localization (25, 26). Knockdown of either the TRAK1 or TRAK2 adaptor significantly reduces the numbers of moving mitochondria in cultured hippocampal axons and dendrites, respectively (17, 26). Currently, however, the molecular nature of other components of the Miro-TRAK machinery remain poorly understood.

Mitochondrial trafficking and morphology are tightly linked (27). Mitochondrial morphology is dependent on the balance of fission and fusion. Fission is regulated by Drp1 (dynamin-related protein 1), which is recruited to the mitochondria by anchors such as Fis1 (mitochondrial fission protein 1). Fusion is coordinated by the GTPases Mitofusin1 and −2 at the outer mitochondrial membrane, which tether two mitochondria together, and OPA1 at the inner membrane (28). These fusion events are necessary for the exchange of mitochondrial contents, *e.g.* mitochondrial DNA and metabolites, maintaining mitochondrial function, and mitochondrial biogenesis (29). Mitofusin2 also plays an important role in bridging mitochondria to the endoplasmic reticulum (ER)^9^ (30). Mitochondria-ER contacts facilitate communication between these two organelles, including the transfer of calcium and lipids (31), and are known sites of autophagosome biogenesis (32). Additionally, contacts between the ER and mitochondria are proposed to be involved in both fission-fusion and the trafficking of mitochondria (33); interestingly, the yeast homologue of Miro1, Gem1, is also known to be localized to these sites (34). However the role of Miro in pathology at Mito-ER contacts is unclear.

DISC1 can be found localized to mitochondria (35, 36) and has been demonstrated previously to modulate the function and transport of mitochondria and other key cargo in neuronal axons (35, 37–39), whereas disease-associated DISC1 point mutations lead to disrupted mitochondrial trafficking (39, 40). Although DISC1 appears to be important for mitochondrial trafficking in neuronal axons, whether DISC1 also impacts mitochondrial trafficking in dendrites, a key locus for altered neuronal function in schizophrenia and other major mental illness, is unknown. Moreover, the mechanisms by which DISC1 regulates mitochondrial trafficking and the impact of this regulation on neuronal development remain unclear. Additionally, the effects of the schizophrenia-associated DISC1-Boymaw fusion protein on mitochondrial dynamics and neuronal development are also poorly understood.

Here we explored further the role that DISC1 plays in mitochondrial dynamics, addressing the interactions of DISC1 with mitochondrial trafficking complexes and fusion machinery. We investigated effects of DISC1 on mitochondrial trafficking in dendrites and subsequent actions on dendritic development. Using biochemical assays together with live cell imaging experiments, we have demonstrated that DISC1 forms protein complexes with the dendritic TRAK2/Miro trafficking complex and with the mitofusins. We biochemically mapped the interaction among Miro, TRAK, and DISC1 to the DISC1 N terminus, demonstrating that overexpression of the Miro N-terminal binding domain of DISC1 disrupts both mitochondrial dynamics and dendritic development in neurons. Furthermore, we have shown that the DISC1-Boymaw fusion protein, resulting from the chromosomal translocation described in a Scottish pedigree (4), acts in a dominant negative fashion, significantly impairing mitochondrial dynamics, the mitochondria-ER interface, and dendritic morphogenesis.

## Materials and Methods

### 

#### 

##### Antibodies and Constructs

Antibody against neurofascin (clone A12/18) was from NeuroMab (IC 1:100). Antibody against GFP was from Santa Cruz Biotechnology (Western blot 1:500, sc-8334) or NeuroMab (clone N86/8). For Sholl analysis, anti-GFP antibody was from Nacalai Tesque, Inc. (1:2000, G090R). The monoclonal antibodies 9E10 (recognizing Myc) and 12CA5 (recognizing HA) were obtained from respective hybridomas (Western blot and immunofluorescence 1:100, mouse). Anti-human DISC1 (14F2) was described previously (WB and immunofluorescence 1:100 mouse) (41); anti-Rhot1 against Miro was from Atlas Antibodies (HPA010687 for proximity ligation assays and AMAb90854 for immunoprecipitation), and anti-TRAK1 was also from Atlas Antibodies (HPA005853). Mitofusin1 was from Abcam (Ab57602), and TOM20 was from Santa Cruz Biotechnology (FL-145). Secondary antibodies for immunofluorescence were from Invitrogen and were used at 1:1000. Secondary horseradish peroxidase-conjugated antibodies were from Rockland and used at 1:10,000. The cDNA construct encoding human ^myc^DISC1-FL was a kind gift from N. Brandon (Cambridge, MA). Untagged human DISC1 was in a pRK5 expression vector (42). Mitochondrially targeted monomeric DsRed fluorescent protein (MtDsRed2), synaptophysin^GFP^, ^GFP^Miro1, and ^GFP^Miro2 were described previously (17, 20, 21, 43). Endoplasmic reticulum-targeted DsRed fluorescent protein (ERDsRed) was from Clontech. ^GFP^TRAK1 and ^GFP^TRAK2 were cloned by insertion of the mouse TRAK sequences into the EGFP-C1 vector. HA-tagged DISC1 deletion constructs were described previously (44). ^Myc^TRAK constructs were a kind gift from F. A. Stephenson (University College London School of Pharmacy). TRAK Miro binding domain (MBD) was described previously (23). ^HA^Boymaw, a kind gift from M. Geyer (University of California, San Diego), was subcloned into the pRK5 expression vector (6). The following constructs were from Addgene: ^myc^Mitofusin1 (plasmid 23212) and -2 (23213), Su9-EGFP-(23214) (45), and mito-PAGFP-(23348) (46).

##### Cell Culture and Transfection

COS7 and SH-SY5Y cells were maintained in 10-cm dishes containing 10 ml of enhanced DMEM supplemented with penicillin-streptomycin and 10% FBS at 37 °C and 5% CO_2_, transfected by nucleofection using an Amaxa electroporator, and allowed 24–48 h for protein expression. For preparation of the primary neuronal cultures, embryonic day 18 (E18) pups were removed from the dam under sterile conditions. Brains were removed from the skulls, and hippocampal dissection was carried out in Hanks' balanced salt solution at 4 °C prior to incubation in a 0.125% trypsin-EDTA solution for 15 min at 37 °C. Hippocampi were washed three times with 10 ml of Hanks' balanced salt solution and triturated 10 times using a fire-polished Pasteur pipette in prewarmed attachment medium. Cells were plated at 350,000 cells in 5 ml of prewarmed attachment medium (minimum Eagle's medium plus 10% horse serum) in 6-cm dishes containing washed glass coverslips precoated overnight in 500 μg/ml poly-l-lysine. After 5 h the medium was removed and replaced with prewarmed maintenance medium (Neurobasal medium supplemented with 2% B-27 (Gibco), 6% glucose, GlutaMAX, and penicillin-streptomycin). Calcium phosphate precipitation or lipofection methods were used for transfection of hippocampal cultures at 7 days *in vitro* (DIV) for Sholl analysis or 8 DIV for live imaging. For calcium phosphate, 1–2 μg of DNA was prepared in 27 μl of Tris-EDTA, 3 μl of 2.5 m CaCl_2_, and 30 μl of 2× HEPES-buffered saline. Coverslips were treated in 1 ml of prewarmed unsupplemented Neurobasal medium with the calcium phosphate preparation. The dishes were then returned to the 37 °C, 5% CO_2_ incubator for 30 min or until a fine precipitate was formed. Coverslips were washed twice, and samples were maintained in the original conditioned medium for 24–48 h for live imaging or 72 h for Sholl analysis in the 37 °C, 5% CO_2_ incubator to allow expression of the transfected vectors. Lipofection was carried out according to the manufacturer's instructions (Invitrogen) in unsupplemented Neurobasal medium with 6% glucose.

##### Biochemical Assays

Co- immunoprecipitation experiments were carried out in lysis buffer (50 mm HEPES, pH7.5, 0.5% Triton X-100, 150 mm NaCl, 1 mm EDTA, 1 mm PMSF, and 1 μg/ml antipain, pepstatin, and leupeptin) using GFP trap beads (Chromotek) or rabbit anti-Myc beads (Sigma). For native co-immunoprecipitation experiments, the brains of transgenic rats expressing full-length, non-mutant, human DISC1 were used (80). Co-immunoprecipitation (co-IP) was carried out in lysis buffer with 1.5% Triton X-100. The homogenate was incubated overnight with antibodies in the above described buffer supplemented with 1% BSA. Protein A-beads (Sigma) were used, and IPs were washed four times in incubation buffer and once in lysis buffer.

##### Western Blotting

SDS-PAGE and Western blotting samples were denatured at 94 °C for 5 min in 3× SDS sample buffer (150 mm Tris, pH 8, 6% SDS, 0.3 m DTT, 0.3% bromphenol blue, and 30% glycerol). Polyacrylamide gels were prepared using 10% running gels and 5% stacking gels in Novex 1.5-mm cassettes and run using the Novex XCell SureLock Mini-Cell system. Gels were transferred onto Hybond-C nitrocellulose membrane (GE Healthcare). Membranes were blocked in 4% milk for 1 h and incubated overnight at 4 °C with shaking in the appropriate antibody. HRP-conjugated secondary antibodies were from Rockland (1:10,000). Bands were visualized using Crescendo chemiluminescent substrate (Millipore) together with an ImageQuant LAS 4000 charge-coupled device camera system (CCD, GE Healthcare).

##### Immunocytochemistry

**FIGURE 1.**
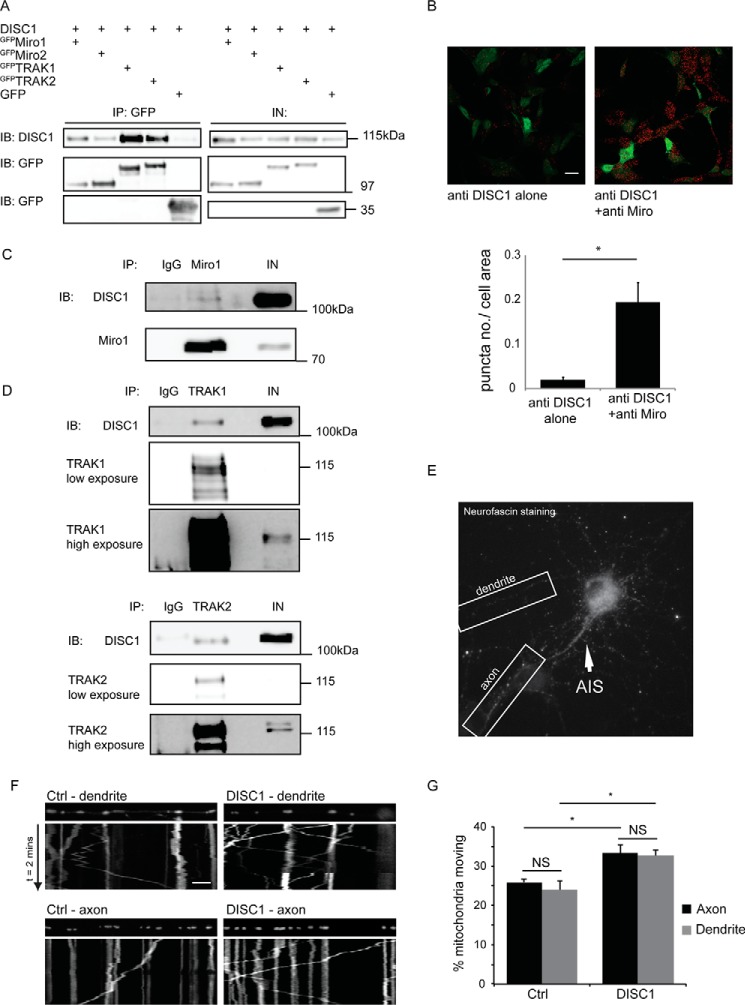
**DISC1 interacts with mitochondrial trafficking complex proteins to regulate transport in dendrites in addition to axons.** *A*, GFP trap co-immunoprecipitation experiments from COS7 cells show robust interaction of DISC1 with ^GFP^Miro1, ^GFP^Miro2, ^GFP^TRAK1, and ^GFP^TRAK2. *B*, proximity ligation assay in SH-SY5Y cells with DISC1 antibody or a DISC1 and Miro1 antibody shows significantly increased signal in the dual antibody condition over background, indicating that DISC1 and Miro1 interact within the cell (*n* = 3 individual preparations). *Scale bar* = 20 μm. *IB*, immunoblot; *IN*, input. *C* and *D*, co-immunoprecipitation experiments with rat brain homogenate showing DISC1 to be part of a native complex with Miro (*C*) and TRAK1 (*n* = 3) or TRAK2 (*n* = 3) (*D*). *E*, example of live labeling of the axon initial segment (*AIS*) to distinguish axons and dendrites within the same neuron. *F*, kymographs showing movement of mitochondria through the axons and dendrites over time. Moving mitochondria are indicated by *diagonal lines* and stationary mitochondria by *straight lines. G*, dendritic compartments (*gray bars*) and axonal compartments (*black bars*) were assayed for mitochondrial movement, and the percentage of moving mitochondria was quantified with and without expression of DISC1 (dendrites: *n* = 15 control neurons and *n* = 16 DISC1 neurons, *, *p* = 0.02; axons: *n* = 16 control neurons and *n* = 13 DISC1 neurons, *, *p* = 0.02). *Scale bar* = 5 μm. *NS*, not significant.

As shown in Fig. 1, fluorescent labeling was used during live imaging to determine the axonal compartment prior to mitochondrial imaging (47). Thus, neurofascin antibody (1 μl) was incubated for 15 min on ice with the secondary fluorescently conjugated antibody (0.3 μl). 100 μl of live imaging block solution was added (10% horse serum and 90% extracellular solution), the solution was mixed, and a coverslip containing the cells for imaging was incubated in this mixture for 8 min at room temperature. Following one rinse in 1× PBS, the coverslip was used for imaging as described below. Fixed cell imaging was carried out by fixation with 4% paraformaldehyde for 10 min at room temperature followed by blocking for 10 min in 10% horse serum, 0.5% BSA, and 0.2% Triton X-100 in PBS. Coverslips were incubated in the relevant primary antibodies diluted in blocking solution for 1 h, washed five times in PBS, and incubated in secondary antibodies diluted in blocking solution. Coverslips were washed five times in PBS, mounted onto slides using Prolong® Gold antifade reagent (Invitrogen), and later sealed with nail varnish. Proximity ligation assays (Duolink) were carried out using anti-Rhot1 (HPA010687) and anti-DISC1 antibodies (14F2 both 1:200) or anti-DISC1 alone for control proximity ligation assays. Samples were fixed and blocked before primary antibodies against Miro1 (Rhot1) and DISC1 raised in either mouse or rabbit were applied to the cells. Following primary antibody incubation, cells were washed in PBS before incubation with secondary antibodies conjugated with oligonucleotides. Ligation and amplification reactions were conducted at 37 °C, as described in the Duolink manual, before mounting and visualization using confocal microscopy (48). Cell fusion assays were carried out as described previously (45). Briefly, cells were nucleofected with MtDsRed2 or Su9-EGFP and plated together. 24 h later, the medium was replaced with 50% polyethylene glycol 1500 in unsupplemented DMEM for 45 s and washed three times every 10 min. Normal medium was replaced and supplemented with 30 μg/ml cycloheximide. Cells were fixed and imaged 3 h later. Imaging was carried out using a Zeiss LSM 700 upright confocal microscope using an Apochromat 63× oil immersion lens with a 1.4 numerical aperture. Images were captured digitally using Zen 2010 software. For ER-mitochondria contact analysis, post-acquisition processing on stacks was carried out in ImageJ using denoise and deconvolution plugins (49, 50) followed by a three-dimensional rendering with VolumeJ. Images of ER-mitochondria contacts were generated using the “image calculator” function of ImageJ to generate images specifically of colocalized regions. Structured illumination microscopy (SIM) was performed using a Zeiss Elyra PS.1 equipped with 405-, 488-, 555-, and 642-nm lasers. Images were acquired with a 63 × 1.4 numerical aperture oil immersion objective using a pco.edge sCMOS camera and Zen 2012 image analysis software. Typically, images were acquired with 34-μm grating and three rotations by exciting fluorophores with 1–3% laser intensity and 120–150-ms exposure time. Post-acquisition, images were processed with Zen 2012 using the SIM reconstruction module with default settings; drift corrections between the channels were performed with respect to 100-nm Tetraspec fluorescent microspheres (Molecular Probes). For Sholl analysis an apochromat 40× oil immersion lens with a 1.3 numerical aperture was used. Neurites were traced in NeuronStudio. The number of intersections was calculated using the neurite tracer plugin on ImageJ (51).

##### Live Cell Imaging

For neuronal imaging of the mitochondria, E18 primary hippocampal neurons were transfected at 7–8 DIV, imaged at 9–10 DIV under perfusion with imaging medium (125 mm NaCl, 10 mm HEPES, 10 mm glucose, 5 mm KCl, 2 mm CaCl_2_, and 1 mm MgCl_2_, pH 7.4) warmed to 37 °C, and flowed at a rate of 1–2 ml/min throughout the duration of each experiment (52). For acquisition, fluorescence was captured using an Olympus microscope (BX60M) with a 60× Olympus objective coupled to an EM-CCD camera (iXon, Andor Technology). Excitation was provided by a mercury arc lamp (Cairn Research) with the appropriate filters (53). Images were acquired at 1 frame/s for a period of 2 min. Axonal regions were acquired at a distance of 100–200 μm from the cell body, and dendritic imaging was acquired at a distance of 50 μm from the cell body due to their reduced length. The length of process assayed was ≈150 μm. To create kymographs, image sequences were opened within ImageJ. Curved processes were straightened using the “straighten” macro, and kymographs were created using the “multiple kymograph” macro. The resulting kymographs show the process along the *x* axis and time across the *y* axis. Mobility was assessed by counting the percentage of objects moving during an imaging period. Mitochondria and synaptophysin^GFP^ positive vesicles were classed as moving if they moved more than 2 μm between the initial and final frames of acquisition (17). Photoactivation assays were carried out on a Zeiss LSM 700 upright confocal microscope using an apochromat 60× water immersion lens with a 1.0 numerical aperture. Photoactivation was carried out at 405 nm after five frames, and spread of GFP signal was measured in ImageJ over 95 frames at 1 frame/6 s.

##### Statistical Analysis

All data were obtained using cells from three different preparations unless otherwise stated. Individual differences were assessed using individual Student's *t* tests at a 95% significance level. Statistical significance across groups was analyzed using one-way analysis of variance and Tukey's post hoc test to compare all data groups. For Sholl analysis, we used two-way repeated measures analysis of variance with a post hoc Bonferroni test for comparison of dendritic crossing and branch points. Data are shown as mean ± S.E. Both the Pearson and Manders coefficients were calculated using the JACoP plugin within ImageJ.

## Results

### 

#### 

##### DISC1 Couples to the TRAK-Miro Trafficking Complex to Affect Mitochondrial Trafficking in Axons and Dendrites

We and others have previously identified DISC1 as a regulator of mitochondrial trafficking in axons via knockdown and overexpression studies (39, 40), and DISC1 was recently shown to interact with TRAK1, a predominantly axonal trafficking protein (39). In contrast, whether DISC1 can interact with the dendritic mitochondrial trafficking adaptor TRAK2 to regulate dendritic trafficking of mitochondria remains unclear. To address this question, we carried out co-IP experiments in COS7 cells co-expressing the mitochondrial trafficking adaptors ^GFP^TRAK1, ^GFP^TRAK2, ^GFP^Miro1 or ^GFP^Miro2, and DISC1. Using GFP-TRAP beads we could readily pull down DISC1 with TRAK1 and TRAK2 and with Miro1 and Miro2 but not with GFP alone (Fig. 1*A*). Conversely, TRAK2 could be robustly pulled down with immunoprecipitated DISC1 (data not shown). Thus, DISC1 interacts robustly with TRAK2 in addition to Miro proteins.

To confirm the interaction between endogenous DISC1 and Miro1 *in situ*, we also carried out proximity ligation assays in SH-SY5Y cells. This assay detects interactions between endogenous proteins in fixed samples, giving a fluorescent readout after incubation with relevant primary antibodies, ligation, and amplification steps (48). The significant increase in puncta in the dual antibody condition indicated an intramolecular distance of <40 nm between DISC1 and Miro1, suggesting that the interaction occurs with endogenous proteins under physiological conditions (Fig. 1*B*). To confirm with this finding, we performed co-IP experiments with rat brain homogenate. We demonstrated the interaction of DISC1 and Miro/TRAK proteins in the brain tissue (Fig. 1, *C* and *D*). These experiments validate DISC1 as a part of the native mitochondrial trafficking complex and so able to mediate its effects locally at the mitochondrion in neurons.

Up-regulating DISC1 levels in neurons increases mitochondrial trafficking in axons (39, 40). The interaction between DISC1 and dendritic TRAK2 led us to explore whether DISC1 could also regulate mitochondrial motility in dendrites. We compared DISC1-mediated trafficking effects on mitochondrial movement in axons *versus* dendrites within the same neurons. Neurons transfected at 8 DIV with MtDsRed2, a mitochondrially targeted red reporter construct, with or without DISC1 were imaged at 9 DIV. DISC1 expression was confirmed by post hoc immunostaining. To unequivocally label axons, the axon initial segment was live labeled with fluorescent anti-neurofascin antibody (47), and the mitochondria were labeled by expressing MtDsRed2 (Fig. 1*E*) (17). We found that in both axonal and dendritic compartments there was an equal enhancement in mitochondrial motility on co-expression of DISC1 (Fig. 1, *F* and *G*, percentage of moving mitochondria in axons; 33.33 ± 2.16% for DISC1, *n* = 13 neurons, compared with 25.87 ± 0.82% for control, *n* = 16 neurons; in dendrites with DISC1, 32.55 ± 1.46% of mitochondria were moving, *n* = 16 neurons, compared with 23.84 ± 2.46% for control, *n* = 15 neurons). Thus, DISC1 interacts with the components of the dendritic transport machinery and can regulate the dendritic trafficking of mitochondria.

**FIGURE 2.**
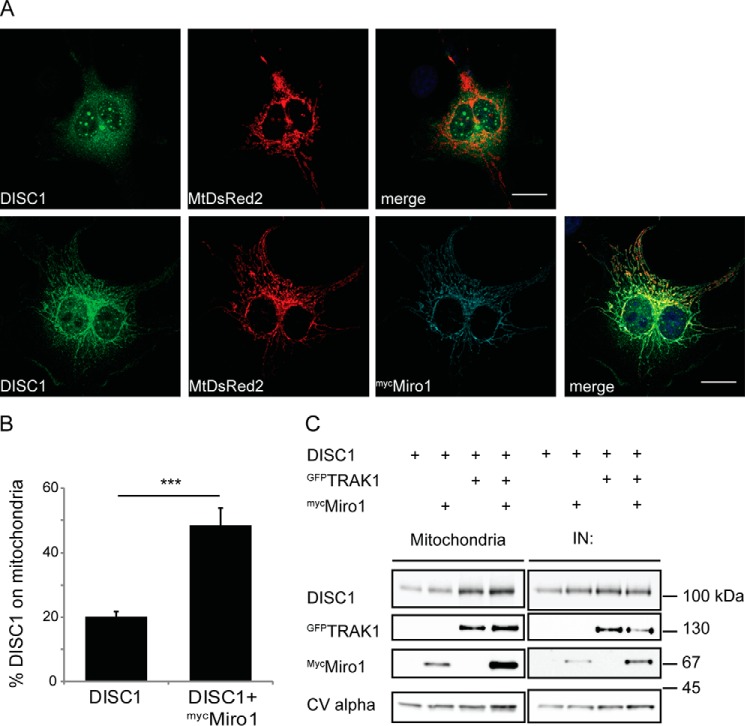
**DISC1 is recruited to mitochondria by components of the mitochondrial trafficking complex.** *A*, immunocytochemistry in COS7 cells showing localization of exogenous DISC1 with and without ^myc^Miro1 overexpression. Mitochondria are labeled with MtDsRed2. *Scale bar* = 20 μm. *B*, percentage of DISC1 on mitochondria shown in *A* (*n* = 13–15 cells from three individual experiments, ***, *p* = 2.29 × 10^−5^). Overexpression of ^myc^Miro1 recruits DISC1 to mitochondria. *C*, mitochondrial fractionation from COS7 cells shows an increase in DISC1 in this compartment with ^GFP^TRAK1 and ^myc^Miro overexpression (*n* = 3). *IN*, input.

Additionally, we investigated the subcellular localization of DISC1. DISC1 has a varied distribution within the cell and has been demonstrated to adopt nuclear, cytosolic, and mitochondrial distributions (35, 54, 55). First, we investigated the subcellular localization of exogenous DISC1 in COS7 cells. Upon expression of DISC1 alone, ∼20% of the DISC1 protein showed mitochondrial localization as determined by colocalization with the mitochondrial marker MtDsRed2. However, upon co-expression of the mitochondrial trafficking protein ^myc^Miro1, this value increased ∼2.5 fold (from 20.1 ± 1.84% to 48.4 ± 5.52%, Fig. 2, *A* and *B*, *n* = 13 control cells and *n* = 15 ^myc^Miro1-expressing cells). The mitochondrial area was unchanged between the two conditions (data not shown). We also carried out mitochondrial fractionation from COS7 cells to determine the levels of mitochondrial DISC1 upon overexpression of ^myc^Miro1 or ^GFP^TRAK1. In agreement with the immunofluorescence data, we observed an increase in the levels of DISC1 in the mitochondrial fraction upon co-expression of ^myc^Miro1 and ^GFP^TRAK1, implying a recruitment of DISC1 to mitochondria and marking these trafficking complex proteins as acceptors for DISC1 on these organelles (Fig. 2*C*).

##### The DISC1 N Terminus Is Critical for Interaction with Miro and TRAKs and for Mitochondrial Trafficking

**FIGURE 3.**
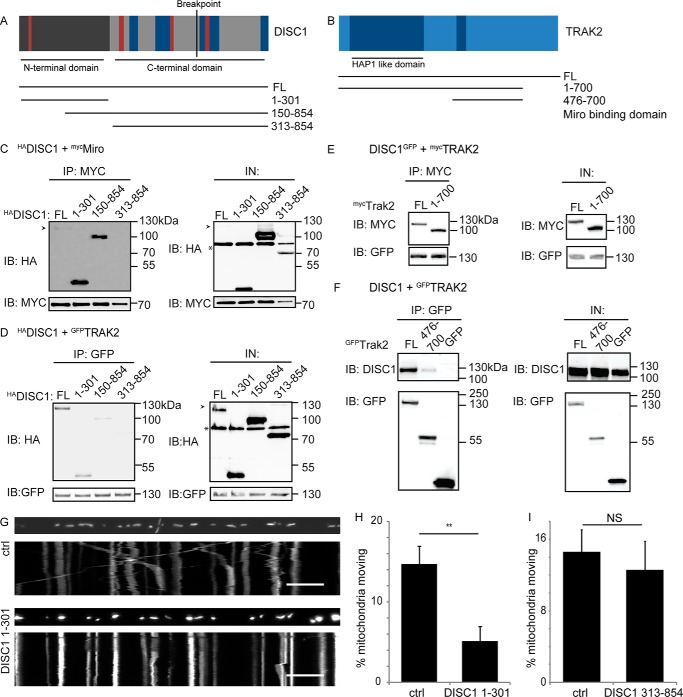
**The DISC1 N terminus mediates the interaction with Miro and TRAKs.** *A*, schematic of the DISC1 protein showing domains present in deletion constructs used. Coiled-coil domains are *dark blue*, and nuclear import or export signals are *dark red. B*, schematic of TRAK2 showing coiled-coil domains (*dark blue*). *C*and *D*, mapping the region of DISC1 that interacts with ^myc^Miro1 (*C*) and ^GFP^TRAK2 (*D*). Co-IP experiments from COS7 cells show that the N-terminal 301 amino acids interact with Miro and TRAK2, whereas amino acids 313–854 are not pulled down. *Arrowhead*, highlights full-length (*FL*) ^HA^DISC1 band; *, indicates nonspecific band. *E* and *F*, mapping the region of TRAK2 which interacts with DISC1. DISC1 interacts with full-length TRAK2 and TRAK2-(1–700) (*E*) but does not interact with the Miro binding domain (*F*). *G*, kymographs showing effect of overexpressing the DISC1-Miro binding domain on mitochondrial transport. *Scale bar* = 10 μm. *H*, percentage of moving mitochondria quantified in axons and dendrites. Overexpression of the DISC1-Miro binding domain prevents mitochondrial transport (*n* = 23 ctrl and *n* = 21 DISC1-(1–301)-expressing neurons from three preparations, **, *p* = 0.002). *I*, quantification of percentage of moving mitochondria in axons and dendrites expressing MtDsRed2 (ctrl) or DISC1-(313–854), the region that does not interact with Miro1 (*n* = 12 ctrl and 11 DISC1-(313–854)-expressing neurons from three preparations, *p* = 0.6). *IB*, immunoblot; *IN*, input; *NS*, not significant.

The molecular determinants of the DISC1 interaction with TRAK1 and TRAK2 remain unclear. DISC1 has a complex structure; it contains a globular N terminus followed by multiple protein-protein interaction domains (36, 56) (Fig. 3*A*). We sought to identify the region of DISC1 that interacts with Miro1 and TRAK2. Co-immunoprecipitation experiments with ^myc^Miro or ^GFP^TRAK2 and ^HA^DISC1 deletion constructs encoding amino acids 1–301, 150–854, and 313–854 of DISC1 showed both the N-terminal 300 amino acids of DISC1 and the longer 150–854-amino acid region as interacting with both Miro1 and TRAK2, whereas the C-terminal coiled-coil-containing region (amino acids 313–854) did not co-IP with Miro or TRAK (Fig. 3, *C* and *D*). Thus, the interaction among DISC1, Miro, and TRAK requires the globular N-terminal domain, likely within amino acids 150–301, with no role for the coiled-coil regions.

We also determined which region of TRAK2, another coiled-coil-rich protein, interacts with DISC1 (Fig. 3*B*). Co-IP experiments were carried out from COS7 cells expressing DISC1 and full-length ^myc^TRAK2 or ^myc^TRAK2-(1–700). TRAK2-(1–700) contains both the Miro binding domain and the N-terminal HAP1-like domain (the site of kinesin binding (23, 24)), as well as one of the binding sites for dynactin subunit p150^glued^ (necessary for dynein motor function) (26). In this case, we saw that both fragments interacted with DISC1 (Fig. 3*E*). Similar experiments with ^GFP^TRAK2 or ^GFP^TRAK2-(476–700), corresponding to the Miro1 binding domain of TRAK2 (23), were also performed. In this case, we saw a marked decrease in the level of DISC1 pulled down with the Miro binding domain of TRAK2-(476–700) in comparison with the full-length protein (Fig. 3*F*). Taken together, these data show that DISC1 binds at the N terminus of TRAK2 (residues 1–476) and not at the Miro1 binding domain. Thus, DISC1 and Miro1 are unlikely to compete for the same interaction site on TRAK, allowing the formation of a functional complex between these three proteins.

To investigate the consequences of disrupting the DISC1-Miro interaction, using live cell imaging we explored the impact of expressing the DISC1-Miro-interacting domain (DISC1 residues 1–301) on mitochondrial transport dynamics. Hippocampal neurons were transfected with MtDsRed2 or co-transfected with MtDsRed2 and ^HA^DISC1-(1–301). Assays were carried out as detailed under “Materials and Methods,” with ^HA^DISC1-(1–301) expression confirmed by immunocytochemistry following live imaging. Upon co-expression of the DISC1-Miro-interacting domain, a significant decrease in moving mitochondria was detected (Fig. 3, *G* and *H*, ctrl = 14.7 ± 2.25%, DISC1-(1–301) = 5.07 ± 1.84%; *n* = 23 control and *n* = 21 DISC1-(1–301)-expressing neurons), as is also apparent by the decrease in diagonal lines in the kymographs. The effect of DISC1-(313–854), which does not interact with Miro, was also investigated and showed no significant alteration in mitochondrial trafficking (quantified data shown in Fig. 3*I*, ctrl = 14.6 ± 2.42%, DISC1-(313–854) = 12.6 ± 3.18%; *n* = 12 control neurons and 11 DISC1-(313–854)-expressing neurons); therefore the impairment in mitochondrial trafficking is reliant on the DISC1-Miro-interacting domain.

##### The Schizophrenia-associated DISC1-Boymaw Fusion Protein Is Localized to Mitochondria and Impairs Mitochondrial Trafficking

**FIGURE 4.**
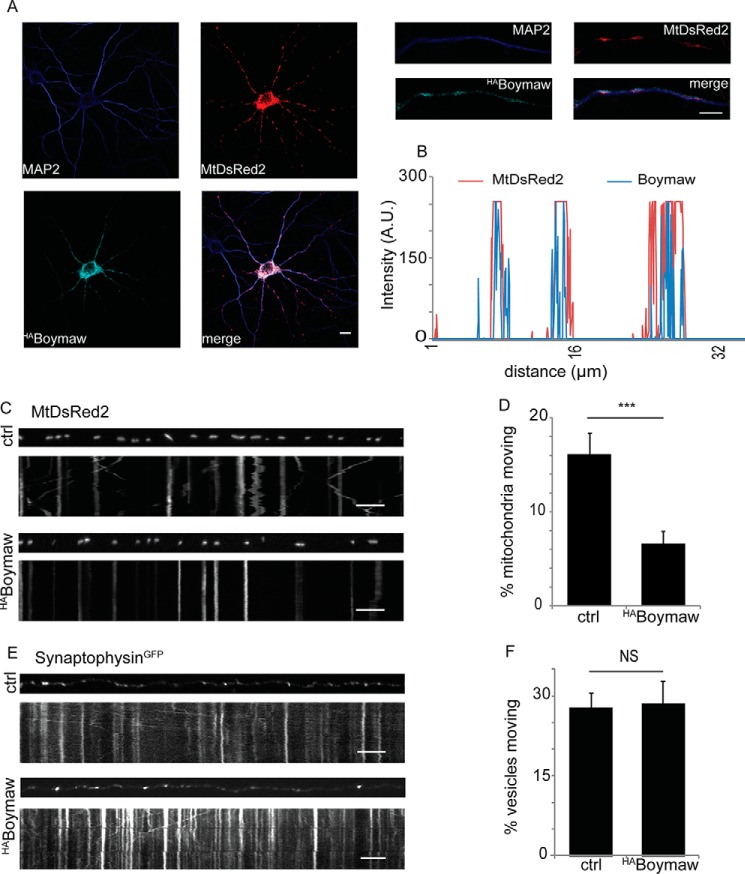
**The DISC1-Boymaw fusion protein inhibits mitochondrial trafficking.** *A*, immunocytochemistry in hippocampal neurons showing localization of the Boymaw protein. *Scale bar* = 20 μm, 5 μm on *zoomed image* (*right*). *B*, line scan of zoomed process showing that the DISC1-Boymaw fusion protein localizes to mitochondria in axons and dendrites. (*A.U.* = arbitrary units.) *C*, kymographs showing mitochondrial transport in axons expressing MtDsRed2 and co-expressing ^HA^Boymaw. *Scale bar* = 10 μm. *D*, percentage of moving mitochondria in neurons was quantified with and without expression of ^HA^Boymaw. The presence of the ^HA^Boymaw fusion protein inhibits mitochondrial trafficking (*n* = 32 ctrl neurons and 26 ^HA^Boymaw-expressing neurons, ***, *p* = 0.001). *E* and *F*, Boymaw expression has no impact on synaptophysin trafficking (*E*), as quantified in *F* (*n* = 17 control and *n* = 19 ^HA^Boymaw-expressing neurons from three preparations, *p* = 0.9). *NS*, not significant. *Scale bar* = 10 μm.

**FIGURE 5.**
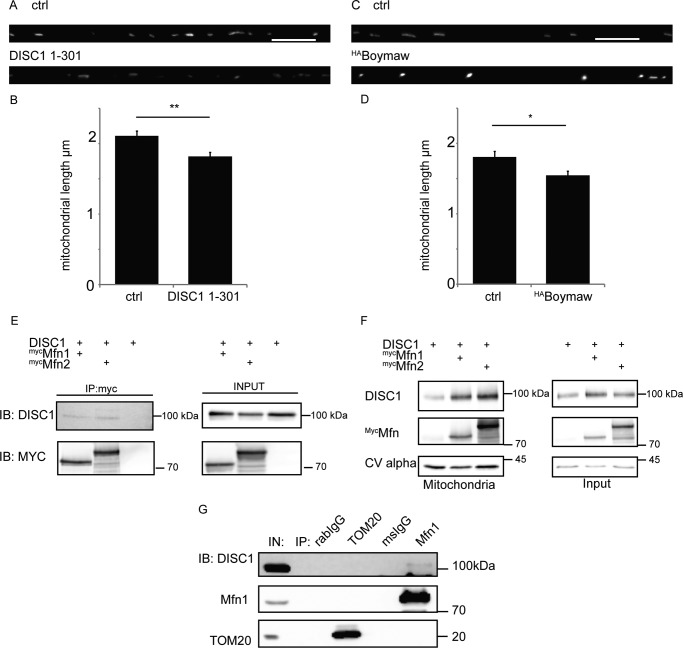
**DISC1 interacts with mitofusins.** *A* and *B*, DISC1 1–301 decreases the length of mitochondria (*A*) as quantified in *B* (ctrl = 2. 1 μm ± 0.065, DISC1-(1–301) = 1.8 ± 0.063 μm, *n* = 11 axons, **, *p* = 0.004). *C* and *D*, the DISC1 Boymaw fusion protein decreases the length of mitochondria (ctrl = 1.81 ± 0.0858 μm, ^HA^Boymaw = 1.54 ± 0.0644 μm, *n* = 13 axons, *, *p* = 0.02). *Scale bar* = 10 μm. *E*, co-IP experiments from COS7 cells show that DISC1 interacts with ^Myc^Mitofusin1 and -2. *F*, mitochondrial fractionation from COS7 cells shows that DISC1 is recruited to mitochondria upon overexpression of ^Myc^Mitofusin1 and -2. *G*, co-IP experiments with rat brain homogenate showing DISC1 to be part of a native complex with Mitofusin1 but not with translocase component TOM20 (*n* = 3). *IB*, immunoblot; *IN*, input.

**FIGURE 6.**
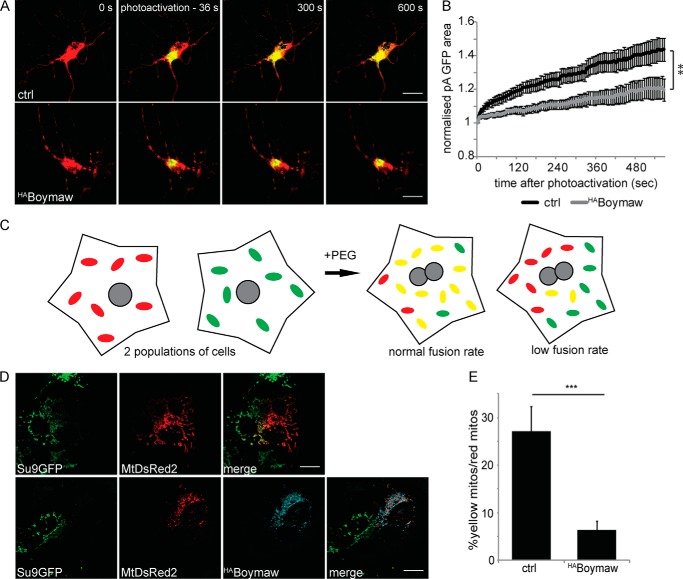
**The DISC1-Boymaw fusion protein inhibits mitochondrial fusion.** *A*, ^HA^Boymaw inhibits mitochondrial fusion in neurons. Neurons were transfected with MtDsRed2 and mitochondrially targeted photoactivatable GFP (ctrl) or coexpressing ^HA^Boymaw. *Scale bar* = 20 μm. *B*, the change in area of GFP signal after photoactivation is reduced in ^HA^Boymaw-expressing neurons (*n* = 17 control and *n* = 15 Boymaw-expressing neurons, *p* < 0.05 at 12–90 s post-photoactivation, **, *p* < 0.01 at 96–570 s post-photoactivation). *C*, schematic showing mitochondrial fusion after polyethylene glycol-mediated plasma membrane fusion. *D* and *E*, ^HA^Boymaw decreases mitochondrial fusion in COS7 cells (*D*) as quantified by colocalization analysis in *E* (*n* = 15 post-fusion cells from three individual preparations, ***, *p* = 0.0009). *Scale bar* = 20 μm.

**FIGURE 7.**
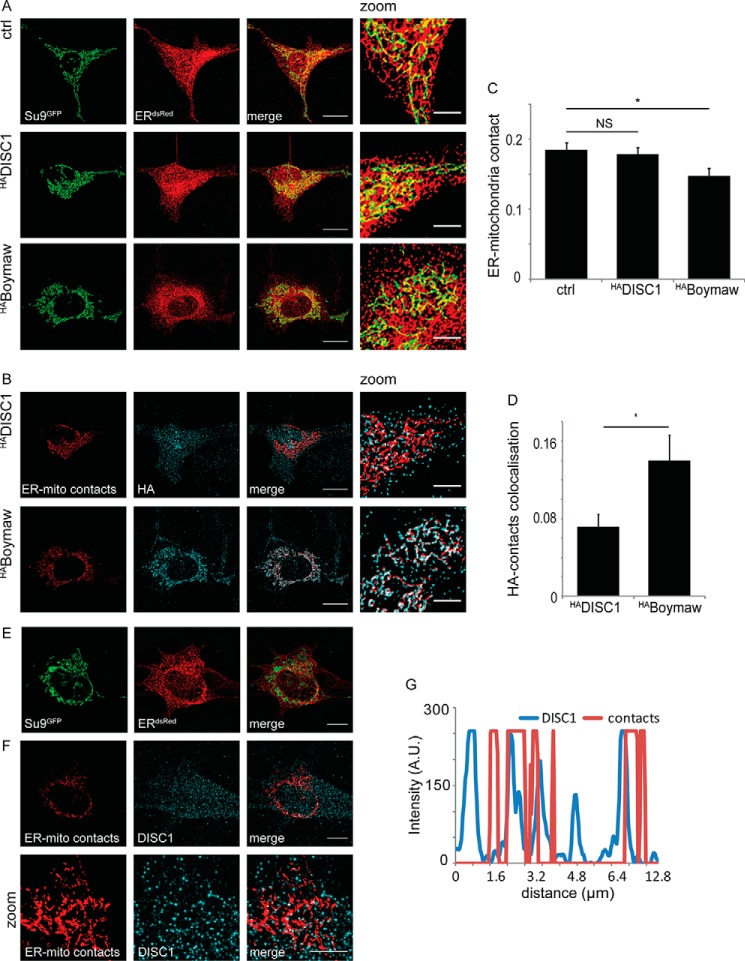
**The DISC1-Boymaw fusion protein decreases the area of ER-mitochondria contacts.** *A*, three-dimensional renderings of mitochondrial network (Su9^GFP^) and ER (ER^dsred^) in COS7 cells upon co expression of ^HA^DISC1 or ^HA^Boymaw. Colocalization shows regions of ER-mitochondria interface. *Scale bar* = 20 μm, 5 μm on *zoomed images* (*right hand panel*). *B*, colocalization of ^HA^DISC1 or ^HA^Boymaw with ER-mitochondria contacts. The images of the contact sites were generated from colocalization of images shown in *A. C*, Manders colocalization coefficient of images in *A* shows a decrease in area of ER-mitochondria contacts upon ^HA^Boymaw expression (*n* = 15 cells from three experiments, ctrl *versus* Boymaw *p* < 0.05). *NS*, not significant. *D*, Manders coefficient for colocalization of ^HA^DISC1 or ^HA^Boymaw with ER-mitochondria contact sites. ^HA^Boymaw shows higher colocalization than ^HA^DISC1 (*, *p* = 0.03). *E*, structured illumination microscopy showing ER and mitochondrial network in SH-SY5Y cells. *F*, structured illumination microscopy shows partial colocalization between endogenous DISC1 and ER-mitochondria contacts in SH-SY5Y cells. *Scale bar* = 10 μm (zoom 1 μm). *G*, line scan showing signal intensities of DISC1 and ER-mitochondria contacts. *A.U.*= arbitrary units.

**FIGURE 8.**
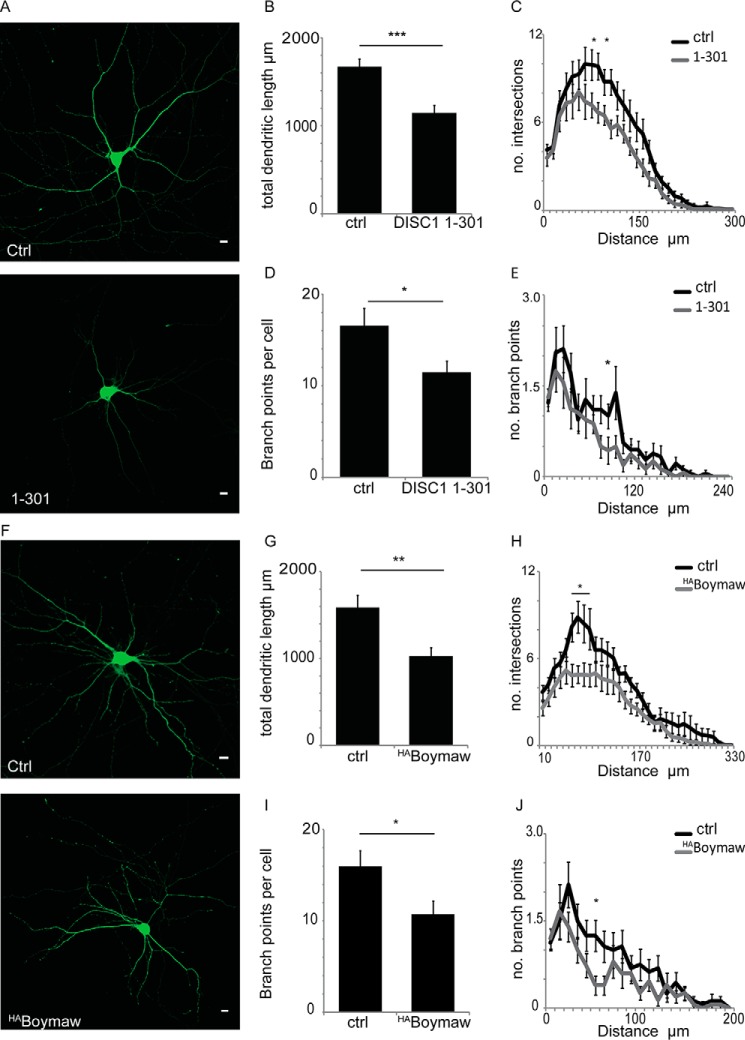
**DISC1-mediated mitochondrial trafficking is necessary for normal dendritic development.** *A*, representative images showing control 10 DIV neurons and those expressing the DISC1-Miro binding domain (residues 1–301). GFP was used to visualize neuronal morphology. *Scale bar* = 10 μm. *B*, total dendritic length/cell (***, *p* = 0.001). *C*, Sholl analysis reveals a significant decrease in intersections at 80 and 100 μm from the soma (*, *p* < 0.05). *D*, average number of branch points/cell is decreased with DISC1-Miro binding domain expression (*, *p* = 0.04). *E*, Sholl analysis of branch points (*, *p* < 0.05 at 90 μm from the soma) (*n* = 16 neurons from four preparations). *F*, representative images showing control 10 DIV neurons expressing GFP in the presence or absence of ^HA^Boymaw. *Scale bar* = 10 μm. *G*, total dendritic length is decreased when the DISC1-Boymaw fusion protein is expressed (**, *p* = 0.004). *H*, Sholl analysis showing the number of intersections at 10-μm intervals. A significant decrease in intersections in Boymaw-expressing neurons is seen proximal to the soma at a distance of 50–80 μm away from the soma (*, *p* < 0.05 in each case). *I*, analysis of branch points reveals expression of the DISC1-Boymaw fusion protein to decrease in the number of branch points/cell (*, *p* = 0.03). *J*, Sholl analysis reveals a significant decrease 50 μm from the soma (*, *p* < 0.05, *n* = 15–16 neurons from four preparations).

The expression of the DISC1-Boymaw fusion protein results from the schizophrenia-associated chromosomal translocation, which interrupts DISC1 in a Scottish pedigree (5, 6). As the fusion protein contains DISC1 amino acids 1–597 (which we show here to include the DISC1-Miro-interacting domain), we investigated the localization of this protein and its effect on mitochondrial trafficking. In hippocampal neurons, the DISC1-Boymaw fusion protein (labeled ^HA^Boymaw in Figs. 4–8) adopts a mitochondrial distribution in neurons as shown by colocalization between MtDsRed2 and ^HA^Boymaw staining seen in the line scan of the zoomed process (Fig. 4, *A* and *B*). Additionally, Pearson colocalization analysis between MtDsRed2 and ^HA^Boymaw gives a coefficient of 0.65 ± 0.08, suggesting a preferential localization to mitochondria. Mitochondrial trafficking assays in neurons transfected with ^HA^Boymaw and MtDsRed2 to label the mitochondria revealed expression of the DISC1-Boymaw fusion protein to significantly decrease the percentage of moving mitochondria compared with control (Fig. 4, *C* and *D*, ctrl = 16.1 ± 2.20%, ^HA^Boymaw = 6.59 ± 1.4%; *n* = 32 control and *n* = 26 ^HA^Boymaw-expressing neurons). In contrast, ^HA^Boymaw expression did not significantly impact the trafficking of synaptophysin^GFP^ positive vesicles (Fig. 4*E*, ctrl = 27.9 ± 2.7%, Boymaw = 28.5 ± 4.3%, *n* = 17–19 neurons; quantified in Fig. 4*F*), confirming that the Boymaw fusion protein is not responsible for an overall decrease in microtubule based transport but specifically disrupts the trafficking of mitochondria. The impact of Boymaw is consistent with the dominant negative effect of the DISC1-Miro-interacting domain on mitochondrial trafficking, suggesting that a disruption in DISC1-mediated mitochondrial trafficking could be a pathological mechanism.

##### DISC1 Couples to the Mitochondrial Fusion Machinery Proteins, Mitofusins

DISC1 had been shown previously to alter mitochondrial morphology (5, 36). Therefore, we measured the length of mitochondria in neurons upon expression of DISC1-(1–301) or the DISC1-Boymaw fusion protein. We found a significant decrease in the length of mitochondria compared with control in each case (Fig. 5, *A* and *B*, ctrl = 2.1 ± 0.065 μm, DISC1-(1–301) = 1.8 ± 0.063 μm, *n* = 11 axons; Fig. 5, *C* and *D*, ctrl = 1.81 ± 0.0858 μm, ^HA^Boymaw = 1.54 ± 0.0644 μm). This observed alteration in mitochondrial morphology led us to investigate the relationship between DISC1 and the mitochondrial fusion machinery. We focused on mitofusins, crucial mediators of mitochondrial fusion and morphology known to interact with Miro proteins (57). Co-immunoprecipitation experiments from COS7 cells expressing ^myc^Mitofusin1 or -2 and human DISC1 revealed that DISC1 could be readily pulled down with both ^myc^Mitofusin1 and -2 (Fig. 5*E*). Moreover, mitochondrial fractionation from COS7 cells confirmed higher levels of mitochondrial DISC1 when either ^myc^Mitofusin1 or -2 was expressed (Fig. 5*F*). Importantly, we confirmed a biochemical interaction between DISC1 and Mitofusin1 from rat brain homogenate (Fig. 5*G*), showing no interaction with the outer mitochondrial membrane protein TOM20 (translocase of the outer membrane of 20 kDa), a protein unrelated to mitochondrial trafficking (58). This shows that DISC1 forms a specific, native complex with fusion proteins rather than interacting indiscriminately with outer mitochondrial membrane proteins. Therefore, DISC1 may play a role in mitochondrial fission/fusion dynamics as well as in trafficking.

##### The DISC1-Boymaw Fusion Protein Decreases Mitochondrial Fusion

Given the interaction between DISC1 and the mitofusins, as well as the effect of the DISC1-Boymaw fusion protein on mitochondrial morphology, we also investigated the impact of Boymaw expression on mitochondrial fusion in primary hippocampal neurons. We used a mitochondrially targeted, photoactivatable GFP (46, 59) as the control condition with co-expression of ^HA^Boymaw and MtDsRed2 expression to visualize the neurons prior to photoactivation. We carried out photoactivation in the neuronal soma, a location of high mitochondrial density and therefore fusion events, to minimize the contribution of trafficking defects to any mitochondrial fusion alteration (27). A decrease in the spread of GFP signal post-photoactivation is seen in ^HA^Boymaw-expressing neurons, showing a decreased mitochondrial fusion rate (Fig. 6, *A* and *B*) (*n* = 17 control and 15 ^HA^Boymaw neurons, final normalized area ctrl = 1.44, ^HA^Boymaw = 1.20 arbitrary units).

Although this photoactivation assay gives an indication of the fusion rate independent from mitochondrial trafficking, there is the potential for contribution of the reported trafficking defect to the decreased spread of GFP signal. To confirm our results by another method, we used a previously described assay (45) involving transfection and co-culture of two populations of cells (in this case COS7 cells) with different mitochondrial markers (in this case fluorophores Su9^GFP^ and MtDsRed2 or with ^HA^Boymaw co-expressed with MtDsRed2) followed by PEG 1500 treatment to fuse the plasma membranes after 24 h. paraformaldehyde fixation and immunostaining was carried out 3 h later (see schematic in Fig. 6*C*). This assay has the advantage of mitochondrial ^HA^Boymaw expression in just half of the cells, and therefore the trafficking of Su9^GFP^ positive mitochondria is unimpeded and fusion can be investigated with a lesser contribution of Boymaw-dependent mitochondrial trafficking deficits. Representative post-fusion cells are shown (Fig. 6*D*), and colocalization of Su9^GFP^ and MtDsRed2 positive mitochondria, indicating fusion events, is quantified (Fig. 6*E*, *n* = 15 fused cells, ctrl = 27% ± 5.2, ^HA^Boymaw = 6.4 ± 1.9%). The noted decrease in colocalization indicates a Boymaw-dependent impairment in mitochondrial fusion in addition to trafficking. This is consistent with Boymaw acting in a dominant negative manner for fusion as well as trafficking, as suggested by the decrease in mitochondrial length caused by both the DISC1-Miro-interacting domain and ^HA^Boymaw expression. Taken together, these data support a role for DISC1 in mitochondrial fusion as well as trafficking.

##### The DISC1-Boymaw Fusion Protein Decreases the ER-Mitochondria Contact Area

The DISC1 interaction with Miro and Mitofusin 2 (Mfn2), known components of ER-mitochondria contact sites in yeast and mammalian cells, respectively (30, 34), prompted us to investigate effects of DISC1 and the DISC1-Boymaw fusion protein on the ER-mitochondria interface. We used COS7 cells because of their extensive ER network and expressed Su9^GFP^ and ER^dsRed^ to label the mitochondria and ER, respectively, along with ^HA^DISC1 or ^HA^Boymaw. Representative volume renderings are shown in Fig. 7*A*. Colocalization analysis between the ER^dsRed^ and Su9^GFP^ signals by Manders coefficient indicates the area of ER-mitochondria contacts, revealing a significant decrease in this area in the presence of ^HA^Boymaw (Fig. 7*C*, Manders coefficients; ctrl = 0.19 ± 0.04, ^HA^DISC1 = 0.18 ± 0.03, ^HA^Boymaw = 0.15 ± 0.05, ctrl *versus* ^HA^Boymaw, *p* = 0.03, ctrl *versus* DISC1 and DISC1 *versus* ^HA^Boymaw, not significant (*NS*), *n* = 15 cells from three experiments). Additionally, we used these colocalized regions, showing ER-mitochondria contacts for colocalization studies with ^HA^DISC1 or ^HA^Boymaw (Fig. 7*B*) by generating images of the ER-mitochondria contacts. Despite the reduced ER-mitochondria interface, we found a greater fraction of the ^HA^Boymaw signal present at contact sites compared with ^HA^DISC1 (Fig. 7*D*, Manders coefficients; ^HA^DISC1 = 0.07 ± 0.01, ^HA^Boymaw = 0.14 ± 0.03, *p* = 0.03). This alteration in ER-mitochondria contact sites suggests potential roles for DISC1 in ER-mitochondria cross-talk and mitophagosome biogenesis (32). To further investigate the potential that DISC1 might be resident at ER-mitochondria contacts, we carried out SIM imaging. This technique gives images with resolution approaching 120–130 nm and thus is of great value in imaging these microdomains (60). SIM imaging was performed in SH-SY5Y cells transfected with Su9^GFP^ and ER^dsRed^ and stained for endogenous DISC1. Fig. 7*E* shows SIM reconstructed images of the mitochondria and ER, and from these images ER-mitochondria contact images were determined as described previously. On the ER-mitochondria image, the DISC1 image was overlaid and represented in Fig. 7*F*. A line scan showing an overlap of signal intensity in DISC1 and ER-mitochondria contact is shown in Fig. 7*G*. Interestingly, we observed that endogenous DISC1 adopts a punctate distribution, as suggested previously (40), and it can be seen that these puncta colocalize in part with the contact sites between the ER and the mitochondria.

##### DISC1-mediated Mitochondrial Trafficking Is Necessary for Normal Dendritic Arborization

DISC1 plays a key role in the regulation of neurite outgrowth both *in vitro* and *in vivo* (11, 61–63), and growing evidence suggests a link between mitochondrial dynamics and dendrite development and complexity (26, 64, 65). The interaction between DISC1 and dendritically targeted TRAK2 prompted us to investigate the effect of disrupting DISC1-mediated mitochondrial dynamics on dendritic development. GFP expression was used to delineate neuronal morphology, and two markers of complexity were analyzed; dendritic length and dendritic branching. Neurons expressing the DISC1-Miro-interacting domain (DISC1-(1–301)) to disrupt mitochondrial trafficking showed a decreased dendritic complexity (Fig. 8*A*). The total dendritic length per cell was decreased 31% compared with control (Fig. 8*B*, ctrl = 1669.4 ± 99.0 μm, DISC1-(1–301) = 1148.6 ± 88.0 μm, *n* = 16 cells from four preparations, *p* = 0.001). Next we carried out a Sholl analysis to study whether dendrite arbor complexity differs as a function of distance from the soma. This showed the decrease in the number of intersections to be pronounced at 80 and 100 μm from the soma (Fig. 8*C*). A similar analysis was then carried out with the number of dendritic branch points per neuron. As with dendritic length, the number of branch points per cell decreased by 31% upon expression of the DISC1-Miro-interacting domain compared with control (Fig. 8, *D* and *E*, ctrl = 16 ± 1.9 branch points, DISC1-(1–301) = 11.5 ± 1.2 branch points), with the effect most noticeable at 90 μm from the soma.

Repeating this analysis with neurons expressing the DISC1-Boymaw fusion protein showed the same effect (Fig. 8*F*). The calculation of total dendritic length reveals a decrease of 35% compared with control (Fig. 8*G*, ctrl = 1590.4 ± 142.7 μm, Boymaw = 1033.3 ± 101.0 μm, *n* = 15–16 neurons from four individual preparations, *p* = 0.004). A significant decrease in the number of dendritic intersections was noted at 50–80 μm from the soma (Fig. 8*H*). As with dendritic length, the total number of branch points was decreased 33% upon expression of the fusion protein (Fig. 8*I*, ctrl = 16 ± 1.7 branch points, Boymaw = 10.7 ± 1.5 branch points). Concurrently, a Sholl analysis revealed this decrease to be most obvious at 50 μm from the soma (Fig. 8*J*). Taken together, these data demonstrate that the expression of the DISC1-Boymaw fusion protein has a severe negative impact on dendritic development, showing this effect to be linked to impairment in the mitochondrial dynamics. These findings provide further evidence for the importance of correct mitochondrial distribution in the development and maintenance of dendritic arbors. Furthermore, our findings support a key role for DISC1 in this process and further dissect the pathways through which this occurs.

## Discussion

Here we demonstrate that the schizophrenia-associated protein, DISC1, interacts with Miro1 and Miro2 as well as TRAK1 and TRAK2 to affect axonal and dendritic transport of mitochondria. We report the interaction of DISC1 with the mitofusins and confirm DISC1 as part of a native complex with trafficking and fusion proteins in brain tissue. We also demonstrate that the schizophrenia-associated DISC1-Boymaw fusion protein acts in a dominant negative fashion to disrupt mitochondrial trafficking and fusion, as well as decreasing the area of ER-mitochondria contacts. Finally, we demonstrate the necessity of DISC1-mediated mitochondrial dynamics for correct neuronal development and dendritic arborization.

We found Miro1 to be a major mitochondrial acceptor for the DISC1 protein, similar to its effect on the TRAK adaptor and the E3 ubiquitin ligase Parkin, which is crucial for mitophagy (23, 43). Via interaction mapping and subsequent trafficking experiments in neurons, we have demonstrated the necessity of the DISC1-Miro-TRAK interaction for normal mitochondrial transport. Our mapping experiments support the interaction between Miro and DISC1 as occurring within amino acids 150–301 of DISC1. Furthermore, the same region of DISC1 interacting with both Miro1 and TRAK suggests that the interaction occurs with one of these proteins via the other, *e.g.* DISC1 interacts with Miro1 via TRAK. Interestingly, the DISC1-TRAK1 interaction was shown to be increased by ∼30% upon expression of the R37W pathological DISC1 mutant (40), consistent with our identification of the TRAK-interacting domain localizing to the DISC1 N terminus. Because our mapping experiments support the idea that the binding site is within amino acids 150–301, this raises the possibility that the R37W mutation may indirectly impact on DISC1 binding to TRAK1 via a conformational change in the DISC1 N terminus.

It is yet to be determined how DISC1 mediates its effects on trafficking. Although emerging evidence suggests differences between the axonal and dendritic regulation of mitochondrial transport (16, 17, 26, 66), it would seem that DISC1 is a factor that is common to trafficking in both compartments. This is supported by our data demonstrating interactions with both TRAK1 and TRAK2 and comparable up-regulation of mitochondrial motility in axons and dendrites. In a recent article investigating DISC1 and mitochondrial trafficking in axons, DISC1 is reported to show a preference for kinesin-mediated anterograde transport (40). DISC1 interacts with kinesin motors (37, 67), raising the possibility of local regulation of motor activity; this is in agreement with our mitochondrial fractionation assays. Indeed, DISC1 has been reported previously to inhibit GSK3β (68), which in turn phosphorylates kinesin light chains, causing uncoupling of motors from cargo (reviewed in Ref. 69). Moreover, DISC1 is known to interact with and inhibit PDE4 (phosphodiesterase 4), both directly and via transcriptional down-regulation in a complex with ATF4 (activating transcription factor 4) (70–72). A subsequent increase in cAMP could activate PKA, thus inhibiting GSK3β and rescuing a decrease in moving mitochondria as has been reported in studies with a cAMP analogue and the PKA activator forskolin (73). The contributions of these pathways, as well as others, will need to be addressed in the future to fully understand the mechanism by which DISC1 regulates mitochondrial trafficking.

We have shown that the schizophrenia-associated DISC1-Boymaw fusion protein, which targets to mitochondria (5), localizes to mitochondria in MAP2 positive dendritic processes. DISC1-Boymaw expression results in a decrease in the length of mitochondria and disrupts mitochondrial trafficking without impairing the trafficking of other cargoes. Although a role for DISC1 in the trafficking of other cargoes (*e.g.* synaptic vesicles) has been demonstrated (38), a specificity for mitochondria is not unexpected given the mitochondrial localization of the Boymaw fusion protein. Moreover, this is consistent with the effect of DISC1 knockdown on mitochondrial trafficking (39) and interruption of the DISC1-Miro complex by expression of the DISC1-Miro-interacting domain. This may provide a disease mechanism for the *t*(1;11) chromosomal translocation (4). Multiple mutations in DISC1 have been reported previously to disrupt mitochondrial trafficking (39, 40), which can also be disrupted upon DISC1 aggresome formation (52). The interruption of mitochondrial trafficking prevents localization of mitochondria at sites of high energy and calcium buffering demand, *e.g.* synapses (17), and a decrease in mitochondria at synapses has been reported previously in a schizophrenic cohort (74).

We also have reported here that the mitofusins interact with DISC1 and have effects on the localization of DISC1 similar to those of Miro1 and the TRAKs, consistent with these proteins being components of the mitochondrial trafficking complex (57). Additionally, expression of both the DISC1-Miro-interacting domain and the DISC1-Boymaw fusion protein leads to alterations in mitochondrial morphology, prompting investigation into mitochondrial fusion events. We found the DISC1-Boymaw fusion protein to display dominant negative activity on mitochondrial fusion in COS7 cells and primary neurons. These data extend our findings, demonstrating DISC1 as a regulator of mitochondrial fission/fusion dynamics in addition to mitochondrial trafficking. The reported effect on fusion will impact the mitochondrial network, as fusion is crucial for the exchange of contents and mitochondrial biogenesis (29). Intriguingly, a role for DISC1 in the mitochondrial fusion pathway suggests its involvement in maintaining the function of the mitochondrial population.

Here, we also investigated the localization of DISC1 and the DISC1-Boymaw fusion protein at sites of ER-mitochondria contact by confocal microscopy and studied the effects of the DISC1-Boymaw fusion protein on these sites. We found that a greater fraction of the DISC1-Boymaw fusion protein is present at these sites in comparison with wild type DISC1. Notably, we also have demonstrated presence of endogenous DISC1 at these sites by super-resolution microscopy. Further, we have demonstrated that Boymaw expression decreases the area of these contacts. This could reflect inhibition of Mitofusin2 tethering activity, in agreement with our mitochondrial fusion assays and consistent with previous reports showing that Mitofusin2 knock-out mouse embryonic fibroblasts (MEFs) have a significantly decreased area of ER-mitochondria colocalization (30). In addition, the role of ER-mitochondria contacts as sites of trafficking and fission/fusion regulation remains an exciting area for future study, and the effects of schizophrenia-associated DISC1 mutants on these contacts could account for alterations in mitochondrial dynamics and turnover downstream of its effects on autophagosome biogenesis (32). Indeed, alterations in the sites of ER-mitochondria contact have been noted in models of neurodegenerative disease such as amyotrophic lateral sclerosis and Parkinson disease (75, 76).

Finally, we have reported evidence of interplay between DISC1 and mitochondrial trafficking, fusion, and ER-mitochondria contacts in dendritic morphogenesis. Both DISC1 and the proteins mediating mitochondrial dynamics have been shown to affect neurite development. The TRAK proteins have been recently reported to regulate neuronal morphology, with knockdown of TRAK2 decreasing dendritic complexity (26). Mitofusin2 is necessary for normal dendritic development in the cerebellum (65), and studies of Mitofusin1 overexpression also show an alteration in dendritic arborization, suggesting that mitochondrial distribution and fission/fusion play a critical role in dendrite development (64). We have demonstrated a decrease in dendritic complexity upon expression of the DISC1-Miro-interacting domain and the Boymaw fusion protein. Variations in dendritic morphology have been reported previously in models of neuropsychiatric illness, and alteration of DISC1 function, either by truncation or point mutation, is known to decrease neuronal complexity (10, 12, 51, 77). This impairment in dendritic development could lead to disruptions in network connectivity and neurotransmission, leading to the development of schizophrenic symptoms. Indeed, mice expressing Boymaw exhibit behavioral abnormalities such as increased startle and anhedonia, consistent with schizophrenia and depression (78). Collectively, our data support a mechanism whereby impaired DISC1 function leads to aberrant mitochondrial dynamics and dendritic morphogenesis, a causative factor in schizophrenia and other major mental illness.

## Author Contributions

R. N., S. M., T. A. A., W. D. H., and J. T. K. designed the research. R. N., S. M., T. A. A., N. B., D. I., and M. P. performed and analyzed the research. S. V. T. and C. K. generated the tgDISC1 rat and C. K. provided DISC1 antibody. R. N., T. A. A., and J. T. K. wrote the paper.

## Footnotes

9 The abbreviations used are:

ER: endoplasmic reticulum
DIV: days *in vitro*
IP: immunoprecipitation or immunoprecipitate
SIM: structured illumination microscopy
ctrl: control.
